# Antimicrobial susceptibility and distribution trends among inducible AmpC-producing Enterobacterales at a regional health authority in British Columbia: a 5-year retrospective review

**DOI:** 10.1099/acmi.0.001122.v3

**Published:** 2026-04-10

**Authors:** Calvin Ka-Fung Lo, Ahmad Aloufi, Geneviève Amaral, Hasan Hamze, Stephanie May, Jennifer M. Grant, Victor Tsun Ho Yuen

**Affiliations:** 1 Department of Pathology and Laboratory Medicine, University of British Columbia, Vancouver, BC, Canada; 2 Antimicrobial Stewardship Program, Providence Health Care, Vancouver, BC, Canada; 3 Department of Pathology and Laboratory Medicine, Royal Jubilee Hospital, Vancouver Island Health, Victoria, BC, Canada; 4 BC Centre for Disease Control, Public Health Laboratory, Vancouver, BC, Canada; 5 Division of Infectious Diseases and Medical Microbiology, University of British Columbia, Vancouver, BC, Canada

**Keywords:** AmpC, antimicrobial resistance, antimicrobial susceptibility, cefepime, *Enterobacterales*, MIC

## Abstract

**Introduction.** Existing literature supports using cefepime as a carbapenem-sparing agent to treat AmpC-producing *Enterobacterales* infections. To characterize existing susceptibility trends, we conducted a 5-year retrospective study assessing MIC distributions for ceftriaxone, cefepime, piperacillin–tazobactam and meropenem across Vancouver Island Health Authority (Vancouver Island, British Columbia, Canada).

**Methods.** MIC data for AmpC isolates between November 2019 and October 2024 were retrieved (BD EpiCenter™). Blood cultures, invasive specimens, urine and miscellaneous samples (e.g. respiratory and wounds) were included; we excluded surveillance specimens. MIC values were interpreted based upon CLSI M100-E34 breakpoints (Table 2a-1). Descriptive statistics were computed and compared between moderate-risk AmpC (MRAC) inducers versus low-risk AmpC (LRAC) inducers.

**Results.** A total of 5,631 isolates were analysed, with 3,778 (67.1%) identified as MRAC. Across all organisms, susceptibility rates for ceftriaxone, piperacillin–tazobactam, cefepime and meropenem were 82.0%, 87.0%, 95.4% and 98.7%, respectively. Compared to LRAC, MRAC organisms demonstrated lower susceptibility to ceftriaxone (76.0% versus 94.2%) and piperacillin–tazobactam (81.3% versus 98.6%). Cefepime and meropenem demonstrated similar susceptibility rates between LRAC and MRAC. MIC_90_ for cefepime was 1 µg ml^−1^ for all species except *Enterobacter cloacae* (MIC_90_ 4 µg ml^−1^).

**Conclusion.** Between 2019 and 2024, 95.4% of our isolates demonstrated susceptibility to cefepime within our local health authority. Further research will correlate patient outcomes and establish MIC thresholds to guide routine testing and clinical use of cefepime.

## Data Summary

All data associated with this work is reported within the article and supplementary materials.

## Background

AmpC is an Ambler class C *β*-lactamase produced by several *Enterobacterales* species, either due to chromosomal (inducible vs. non-inducible) or plasmid-mediated (usually constitutive) mechanisms. Inducible *AmpC* carriers can pose treatment dilemmas. In wild-type strains, *AmpC* is repressed by *AmpR* via the *AmpD* recycling pathway. Therefore, wild-type isolates display phenotypic susceptibility to third-generation cephalosporins (3GCs) and ureidopenicillins (piperacillin). However, monotherapy exposure with these agents can induce *AmpC* expression by overwhelming repressor pathways and select for strains with *AmpD* mutations, leading to stable chromosomal de-repression. This ultimately translates to *in vitro* resistance against these antimicrobial agents, with a greater risk for clinical treatment failures [1].

*Enterobacter cloacae*, *Klebsiella aerogenes*, *Citrobacter freundii* and *Hafnia alvei* carry the highest mutation risks for *AmpC* de-repression *in vitro* [2]. Conversely, other AmpC-producing *Enterobacterales* (e.g. *Serratia marcescens*, *Morganella morganii* and *Providencia* spp.) carry a lower risk. In 2019, Kohlmann *et al*. found that *AmpC* de-repression mutation rate is variable between different species along with beta-lactam susceptibility [3]. A Spanish study comparing susceptibilities of *E. cloacae* and *K. aerogenes* clinical isolates also demonstrated greater activity of cefepime against *K. aerogenes* (98%, as compared to 90% for *E. cloacae* isolates) [4].

The Infectious Diseases Society of America (IDSA) released guidance documents in 2024 on the treatment of antimicrobial-resistant gram-negative infections and recommended cefepime as a first-line treatment for infections caused by organisms with moderate-risk *AmpC* (MRAC) de-repression (previously classified as moderate to high-risk), such as *E. cloacae*, *C. freundii* and *K. aerogenes* [5]. Cefepime should be avoided in the treatment of infections where isolates have an MIC of 4–8 µg ml^−1^ [i.e. susceptible dose-dependent (SDD)] and co-production of extended spectrum beta-lactamase (ESBL) enzymes [5].

At present, no randomized controlled trials (RCTs) exist directly comparing cefepime to carbapenems for treating AmpC-producing organisms. Recent meta-analyses and retrospective studies focusing on AmpC-producing *Enterobacterales* bacteraemia suggested that even for bacterial isolates with 3GC resistance, cefepime was comparable to carbapenems in terms of clinical outcome and adverse effect rates [6]. However, higher failure rates were still associated with cefepime when treating organisms with MIC in SDD range or those co-expressing ESBL enzymes [7,11].

RCT data has suggested piperacillin–tazobactam as being a viable alternative for *AmpC* organisms that are 3GC-susceptible [12,13]; ceftriaxone has also been considered in settings of low-risk *AmpC* (LRAC) bacteraemia although this evidence is limited and primarily based on retrospective studies [14,15].

Our study objective is to compare MIC distribution of *AmpC*-producing *Enterobacterales* for commonly used antibiotics and to examine the viability of using cefepime as an option for MRAC and ceftriaxone or piperacillin–tazobactam in the treatment of LRAC from the laboratory perspective.

## Methods

We extracted MIC values from the BD EpiCenter™ system (a middleware data management system that houses susceptibility data transmitted from the automated BD Phoenix™ susceptibility testing system; Becton, Dickinson and Company, Sparks, MD, USA) across bacterial isolates collected and processed in Vancouver Island Health Authority Microbiology Laboratory (Victoria, BC) between the date ranges of 1 November 2019 to 30 October 2024. Isolates from blood cultures, invasive sterile sites (e.g. surgical/biopsy specimens), urines and wounds were eligible. Surveillance and screening specimens were excluded from our study.

Antibiotics of interest were ceftriaxone, cefepime, piperacillin–tazobactam and meropenem. Susceptibility interpretations were based on breakpoints from CLSI M100, Ed34 (Table 2a-1, *Enterobacterales*) [16]. Information regarding ESBL production was obtained, if available. On isolates that demonstrated ceftazidime MIC ≥8 µg ml^−1^ on the BD Phoenix™ automated susceptibility panel (BD Diagnostic Systems, Sparks, MD), ESBL is phenotypically confirmed by commercial double-disc diffusion testing with either MASTDISCS^®^ Combi D63C set (for *Enterobacter* and *Citrobacter* species and for *H. alvei*) or MASTDISCS^®^ Combi D68C set (for *Serratia*, *Providencia* and *Morganella* species) (Mast Group Ltd, Bootle, UK). The D63C set includes cefepime 30 µg disc, plus cefepime 30 µg combined disc with clavulanic acid 10 µg. The D68C set includes a cefpodoxime 10 µg disc, a cefpodoxime 10 µg disc combined with ESBL inhibitor, a cefpodoxime 10 µg disc combined with AmpC inhibitor and a cefpodoxime 10 µg disc combined with both ESBL inhibitor plus AmpC inhibitor.

Our analysis included the following organisms: *C. freundii* complex, *E. cloacae* complex, *Enterobacter (Klebsiella) aerogenes*, *S. marcescens*, *Providencia rettgeri*, *M. morganii* and *H. alve*i. Organisms were stratified based on *AmpC* induction risk: moderate-risk (MRAC; i.e. *C. freundii* complex, *E. cloacae* complex, *K. aerogenes *and *H. alvei*) and LRAC (e.g. *S. marcescens*, *P. rettgeri* and *M. morganii*) [5].

Descriptive statistics were computed for each antimicrobial and bacterial species using Microsoft Excel™. We also computed the MIC_50_ and MIC_90_ for each respective organism and antibiotic combination to characterize any concerning trends suggestive of increased resistance.

## Results

In total, 5,631 isolates were analysed, of which 3,778 (67.1%) were considered MRAC species. *E. cloacae* was the most predominant species among MRAC (44.2%); *M. morganii* was the most common among LRAC (51.2%). In terms of specimen types, ~85% of our isolates were derived from urine, 5% from blood and 5% from musculoskeletal specimens, and the remaining 5% were distributed between respiratory, abdominal and other miscellaneous specimens (e.g. wounds). Table 1 summarizes the key descriptives for our sample population.

**Table 1.** Descriptive summary of included isolates (*n*=5,631), by year

Date range (total no. of isolates)	Nov 2019–Oct 2020 (*n*=878)	Nov 2020–Oct 2021 (*n*=991)	Nov 2021–Oct 2022 (*n*=1,032)	Nov 2022–Oct 2023 (*n*=1,283)	Nov 2023–Oct 2024 (*n*=1,447)
**MRAC inducers, *n***	**605**	**695**	**694**	**858**	**926**
*C. freundii*	199 (32.9%)	205 (20.2%)	233 (22.0%)	277 (20.9%)	246 (16.3%)
*E. cloacae*	248 (41.0%)	334 (32.8%)	272 (25.7%)	376 (28.4%)	438 (29.1%)
*H. alvei*	26 (4.30%)	11 (1.08%)	19 (1.79%)	35 (2.64%)	41 (2.72%)
*K. aerogenes*	132 (21.8%)	145 (14.3%)	170 (16.1%)	170 (12.8%)	201 (13.3%)
**LRAC inducers, *n***	**273**	**296**	**338**	**425**	**521**
*M. morganii*	146 (53.5%)	163 (55.1%)	187 (55.3%)	207 (48.7%)	245 (47.0%)
*P. rettgeri*	47 (17.2%)	43 (14.5%)	61 (18.1%)	87 (20.5%)	96 (18.4%)
*S. marcescens*	80 (29.3%)	90 (30.4%)	90 (26.6%)	131 (30.8%)	180 (34.6%)
**Specimen type**					
Blood	54	50	62	53	62
Urine	813	935	966	1,028	1,034
**Ceftriaxone susceptibility**	81.3%	82.8%	81.7%	81.7%	82.3%
**Piperacillin–tazobactam susceptibility**	86.0%	87.4%	87.1%	86.8%	87.4%
**Cefepime susceptibility**	94.4%	95.1%	95.0%	96.3%	95.8%
**Meropenem susceptibility**	98.7%	98.0%	98.6%	99.3%	98.5%

LRAC, low risk AmpC inducers; MRAC, moderate risk AmpC inducers.

Across our study organisms, 82.0% were susceptible to ceftriaxone (corresponding to MIC ≤1 µg ml^−1^), 87.0% to piperacillin–tazobactam (i.e. MIC ≤8/4 µg ml^−1^), 95.4% to cefepime (i.e. MIC ≤2 µg ml^−1^) and 98.7% to meropenem (i.e. MIC ≤1 µg ml^−1^) (Tables1 and 2ad).

**Table 2.** MIC distribution and MIC_50/90_ for corresponding antimicrobial and organism species (a, ceftriaxone; b, piperacillin–tazobactam; c, cefepime; d, meropenem) Values within the cell represent the number of isolates under the corresponding MIC.

**a (ceftriaxone)**									
**MIC value (µg/mL)**	**Susceptible**		**Intermediate**	**Resistant**					
	0.5	1	2	4	8	16	32	64	
MRAC organisms									
*C. freundii* (*n*=1,161)	888†	16	8	9	200*	5	10	25	
*E. cloacae* (*n*=1,668)	1,153†	42	27	15	349*	4	13	64	
*H. alvei* (*n*=131)	76†	27	5	4	15*	3	0	1	
*K. aerogenes* (*n*=818)	663†	7	1	4	111*	8	11	13	
LRAC organisms									
*M. morganii* (*n*=948)	843†	30*	25	6	38	3	0	3	
*P. rettgeri* (*n*=334)	328*†	3	0	0	3	0	0	0	
*S. marcescens* (*n*=571)	509*†	32	10	5	15	0	0	0	
**b (piperacillin–tazobactam)**									
**MIC value (µg/mL)**	**Susceptible**		**SDD**	**Resistant**					
	4	8	16	32	64	128			
MRAC organisms									
*C. freundii* (*n*=1,161)	933†	55	43	39*	44	46			
*E. cloacae* (*n*=1,668)	1,265†	38	55	56	73	181*			
*H. alvei* (*n*=131)	88†	12	10	8*	7	7			
*K. aerogenes* (*n*=818)	658†	23	20	13	55*	49			
LRAC organisms									
*M. morganii* (*n*=948)	935*†	3	3	3	0	4			
*P. rettgeri* (*n*=334)	329*†	3	0	0	0	2			
*S. marcescens* (*n*=571)	551*†	6	7	2	1	4			
**c (cefepime)**									
**MIC value (µg/mL)**	**Susceptible**			**SDD**		**Resistant**			
	0.5	1	2	4	8	16	32		
MRAC organisms									
*C. freundii* (*n*=1,161)	222	861*†	41	15	3	7	11		
*E. cloacae* (*n*=1,668)	270	1,174†	39	84*	43	17	41		
*H. alvei* (*n*=131)	26	97 *†	1	4	1	1	1		
*K. aerogenes* (*n*=818)	152	640*†	13	8	2	1	2		
LRAC organisms									
*M. morganii* (*n*=948)	195	738*†	9	1	2	0	3		
*P. rettgeri* (*n*=334)	56	273*†	1	2	0	1	1		
*S. marcescens* (*n*=571)	98	465*†	2	4	1	0	1		
**d (meropenem)**									
**MIC value (µg/m^L^)**	**Susceptible**				**Intermediate**	**Resistant**			
	0.125	0.25	0.5	1	2	4	8	16	32
MRAC organisms									
*C. freundii* (*n*=1,161)	1,145*†	8	2	0	2	1	1	1	1
*E. cloacae* (*n*=1,668)	1,533*†	49	33	14	15	8	4	6	5
*H. alvei* (*n*=131)	124*†	1	1	2	4	0	0	0	0
*K. aerogenes* (*n*=818)	758*†	40	9	3	3	3	1	0	0
LRAC organisms									
*M. morganii* (*n*=948)	779†	140*	18	0	3	1	1	1	2
*P. rettgeri* (*n*=334)	303*†	22	2	1	1	3	0	0	1
*S. marcescens* (*n*=571)	553*†	11	2	2	0	1	0	0	2

*MIC_90_.

†MIC_50_.

MRAC, moderate-risk AmpC inducers; SDD, susceptible dose-dependent.

### Moderate-risk *AmpC*

**Fig. 1.**
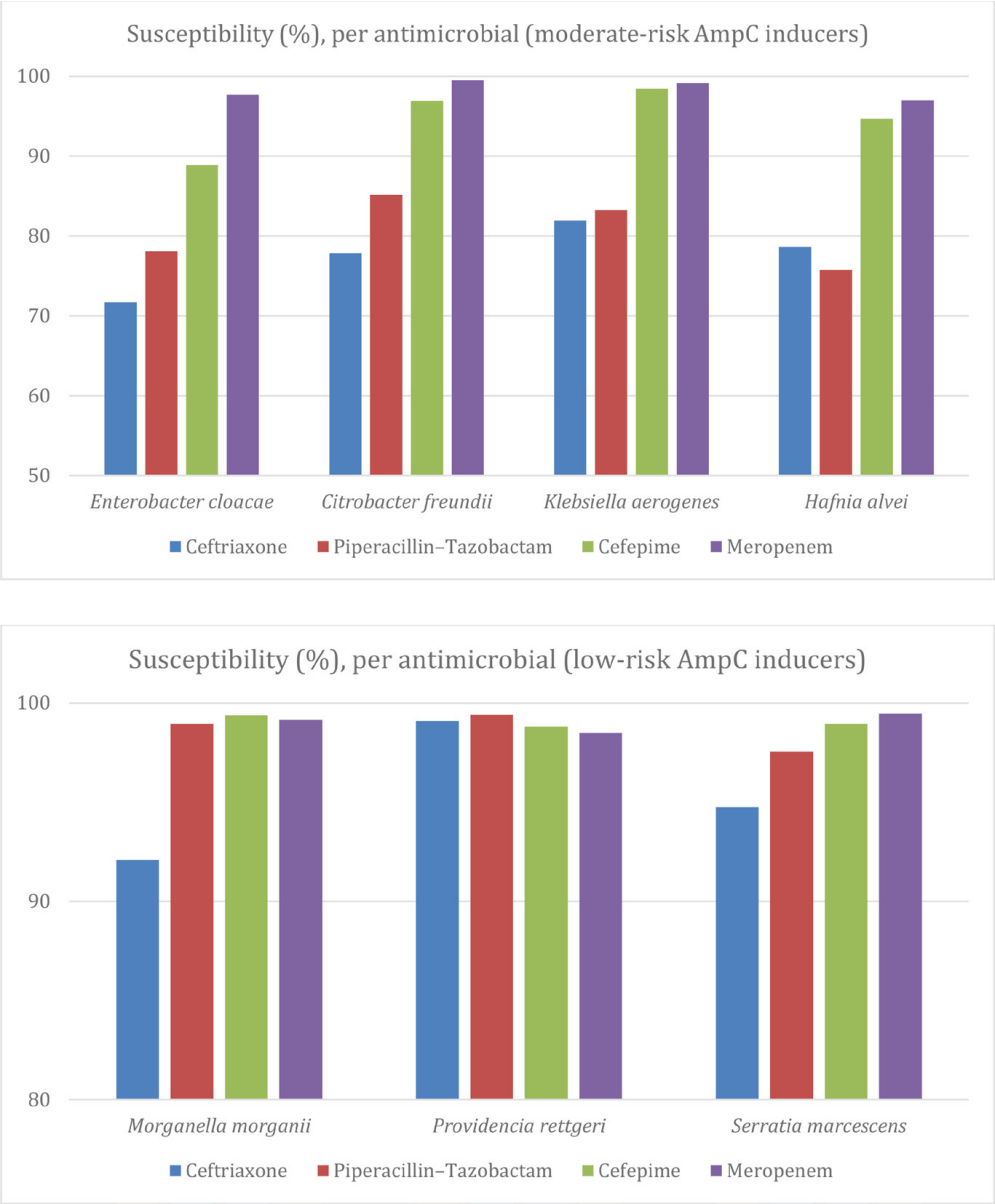
Overall susceptibility of ceftriaxone, piperacillin–tazobactam, cefepime and meropenem, per AmpC induction risk and bacterial species.

MRAC organisms demonstrated lower susceptibility to ceftriaxone (76%) and piperacillin–tazobactam (81.3%). The lowest susceptibility to ceftriaxone was observed among *E. cloacae* (71.6% susceptible) and *H. alvei* (78.6%). Both organisms also had the lowest susceptibility to piperacillin–tazobactam (78.1% for *E. cloacae* and 75.8% for *H. alvei*) (Fig. 1).

Cefepime susceptibility was demonstrated in 93.6% of MRAC organisms, and an additional 4.24% were within the SDD range (MIC 4–8 µg ml^−1^). However, when stratified by species, *E. cloacae* had the lowest susceptibility to cefepime among MRAC organisms (88.9%), compared to 93.9% for *H. alvei*, 96.9% for *C. freundii* and 98.4% for * K. aerogenes*.

Meropenem susceptibility was observed in 98.5% of MRAC organisms.

### Low-risk *AmpC*

LRAC organisms demonstrated 94.2% susceptibility to ceftriaxone and 98.6% to piperacillin–tazobactam overall. When analysed by species, over 90% susceptibility to both antibiotics was observed among LRAC organisms (Fig. 1). Cefepime and meropenem susceptibility were over 98% for LRAC organisms; of note, only ten isolates and six isolates were SDD or resistant, respectively (i.e. <1% total).

### MIC_90_ and MIC_50_

Ceftriaxone MIC_90_ was 8 µg ml^−1^ for all MRAC organisms. For the remaining LRAC species, ceftriaxone MIC_90_ remained relatively low (*M. morganii*, 1 µg ml^−1^; *P. rettgeri* and *S. marcescens*, 0.5 µg ml^−1^). MIC_50_ for all organisms was 0.5 µg ml^−1^.

Piperacillin–tazobactam MIC_90_ was highest for *E. cloacae* (128 µg ml^−1^), *K. aerogenes* (64 µg ml^−1^), then *C. freundii* and *H. alvei* (both at 32 µg ml^−1^). MIC_90_ for the other species (i.e. *M. morganii*, *Proteus vulgaris*, *P. rettgeri* and *S. marcescens*) was 4 µg ml^−1^. MIC_50_ for all organisms was 4 µg ml^−1^.

Cefepime MIC_90_ was 1 µg ml^−1^ across all species except for *E. cloacae* (4 µg ml^−1^). MIC_50_ for all organisms was also 1 µg ml^−1^.

Meropenem MIC_90_ was 0.125 µg ml^−1^ for all species except *M. morganii*, which had an MIC_90_ of 0.25 µg ml^−1^. Specific MIC distributions, MIC_90_ and MIC_50_ for each antibiotic–organism combination are included in Table 2(a–d).

## Discussion

Overall, the 5-year antimicrobial susceptibility trends within our local health authority were stable, although the number of AmpC organisms recovered from submitted specimens increased from <900 AmpC-producing organisms in November 2019–October 2020 to nearly 1,500 organisms by November 2023–October 2024. The proportion of MRAC organisms per year remained between 67 and 70%, except for November 2023–October 2024 where the proportion dropped to 64% although this may be due to an increase in LRAC organisms detected (specifically with *S. marcescens* where 180 isolates were detected in that final year). Annual blood culture counts remained similar across the time period (between 50 and 62 isolates/year), whereas annual urine cultures with AmpC organisms increased from 813 to >1,000/year since November 2022.

Among the organisms in our study, MRAC species had decreased susceptibility to ceftriaxone (76.0%) and piperacillin–tazobactam (81.3%) with *E. cloacae* demonstrating the lowest susceptibility (71.6% for ceftriaxone and 78.1% for piperacillin–tazobactam). It is important to note that regardless of our *in vitro* susceptibility rates being >70% for ceftriaxone, MRAC organisms are well-known to develop resistance after exposure to 3GCs [1,2, 5]. Cefepime had an excellent susceptibility profile: most species were >90% susceptible except for *E. cloacae* (~89%). Our findings align with previous studies [4], which demonstrated that cefepime retained activity for *AmpC* organisms that displayed phenotypic resistance to 3GCs, with the highest resistance seen in *E. cloacae* compared to *K. aerogenes*. On the other hand, meropenem demonstrated >95% susceptibility regardless of species, which is to be expected within *AmpC Enterobacterales* [4].

Among LRAC species, there was >90% susceptibility across all antibiotics, with >98% susceptibility to cefepime and meropenem. Although there remains discussion surrounding induction risk for *H. alvei* [5], our findings indicate that its resistance patterns to ceftriaxone and piperacillin–tazobactam are closely similar to those observed in MRAC species. Such findings are consistent with observations made by Kohlmann [2,3], who noted similar resistance trends and that *H. alvei* had similar mutation rates to other MRAC species.

When compared with other recent literature, we noted that our study data, especially for cefepime, had similar susceptibility percentages; Boattini *et al*. conducted a retrospective survey of over 6,700 isolates and noted >90% susceptibility for cefepime amidst most *Enterobacterales* carrying chromosomal AmpC beta-lactamases except for *E. cloacae* (88% with MIC_90_ of 8 mg l^−1^) and *Providencia* spp. (62.1% with MIC_90_ of 64) [17].

Our study carried several limitations. First, although the sample size was large with over 5,000 isolates, we conducted a single-centre study compiling aggregated laboratory data from Vancouver Island Health Authority rather than the compilation of provincial or national data. Secondly, clinical correlation with treatment decisions and outcomes was not done due to challenges in linking data with our large microbiologic database and the retrospective nature of our study. However, we acknowledge that there could be a selective bias from retrospective study designs even with matching data. For instance, patients who were selected for treatment with cefepime may generally present as more clinically stable, versus a patient in intensive care with septic shock who might be treated with a carbapenem-based regimen. Furthermore, antibiotic choice alone does not necessarily translate to successful outcomes for infections; management is multifactorial and takes into consideration the host status, extent of infection (e.g. localized urinary tract infection versus disseminated multi-focal infection) and source control for infectious foci. Lastly, our extracted data is derived from the BD Phoenix/BD EpiCenter™ system, which is based on automated susceptibility testing for which predicted MICs are generated and manual confirmatory tests were not conducted prior to data extraction.

Considering our findings, we recommend that cefepime susceptibility should be reported for all AmpC-producing organisms, particularly those classified as MRAC. This is vital, as cefepime has demonstrated its effectiveness against many MRAC species [8,11], providing a valuable therapeutic option in managing infections caused by these organisms. Moreover, based on the available limited evidence [12,15], we advocate for the careful reporting of ceftriaxone and piperacillin–tazobactam susceptibility as tested in LRAC cases, while emphasizing the need for ongoing surveillance and research to refine treatment guidelines.

## Conclusion

Our 5-year study demonstrated high rates of cefepime susceptibility (>90%) across AmpC-producing *Enterobacterales* with reassuring MIC distribution for all species. Ceftriaxone and piperacillin–tazobactam should show a similar susceptibility pattern for LRAC species which supports consideration for reporting these antibiotics as tested. Further research is necessary to correlate these findings to patient outcomes and establish MIC thresholds.

## Supplementary material

10.1099/acmi.0.001122.v3 Uncited Supplementary Material 1.

## Abbreviations

3GC: third-generation cephalosporin
LRAC: low-risk AmpC
MRAC: moderate-risk AmpC
RCT: randomized controlled trial
SDD: susceptible dose-dependent
